# Phenolic Compounds in Crop Protection: Mechanisms in Plant–Pathogen Interactions

**DOI:** 10.3390/biology15211911

**Published:** 2026-10-22

**Authors:** Harpreet Kaur, Satinderpal Kaur, Amanpreet Kaur, Swapnika Medikonda, C. Korsi Dumenyo

**Affiliations:** 1 Department of Plant Pathology, Washington State University, IAREC, Prosser, WA 99350, USA; 2 Department of Entomology, University of Illinois Urbana-Champaign, Urbana, IL 61801, USA; 3 Department of Entomology and Plant Pathology, Auburn University, Auburn, AL 36849, USA; 4 School of Food Science, Washington State University, IAREC, Prosser, WA 99350, USA; 5 Department of Agricultural and Environmental Sciences, Tennessee State University, Nashville, TN 37209, USA

**Keywords:** plant diseases, phenolic compounds, cereals, legumes, vegetables, secondary metabolites, plant–pathogen interactions

## Abstract

Plant pathogens cause significant crop yield losses in both agronomic and horticultural ecosystems. During this ongoing evolutionary arms race between plants and pathogens, plants have evolved a diverse array of constitutive and induced defense responses to survive, where secondary metabolites, especially phenolic compounds, are ubiquitous and driven by selective pressures from microbial pathogens. These compounds aid plant health by conferring tolerance and resistance to various biotic and abiotic stresses. Recent advances in omics techniques have provided many ways to extract and quantify these plant compounds and to understand their antimicrobial activity, offering a potential opportunity for sustainable plant disease management strategies. This review emphasizes the role of phenolic compounds as critical secondary metabolites in plant defense and provides a summary across a wide array of agronomic (cereals, legumes, root crops, leafy crops, fodder crops) and horticultural (vegetables, fruits) crops. We explored the distinct mechanisms of phenolic compounds acting as antimicrobials and their differential production in response to pathogenic microbes. We have also highlighted current knowledge gaps to guide future research toward sustainable crop protection by using these compounds in Integrated Disease Management strategies.

## Simple Summary

Plant diseases caused by bacterial and fungal pathogens are major threats to agricultural and horticultural cropping systems. Therefore, improving plants’ ability to defend against pathogens is critical for developing effective and sustainable disease management strategies. Phenolic compounds are important components of plant defense against pathogens. Following pathogen recognition, defense signaling can activate genes involved in phenylpropanoid metabolism, leading to production and accumulation of diverse phenolic compounds that contribute to antimicrobial activity, cell wall reinforcement, redox regulation, and other defense responses. In this review, we highlight different types of phenolic compounds found in agricultural and horticultural crop species and their role in defending against the corresponding plant pathogens. We argue that advances in the extraction and characterization of individual phenolic compounds have improved our understanding of their diverse roles in plant defense. Future research should focus on integrating this knowledge into breeding programs to develop crops with improved resistance to pathogens.

## 1. Introduction

**Figure 1:**
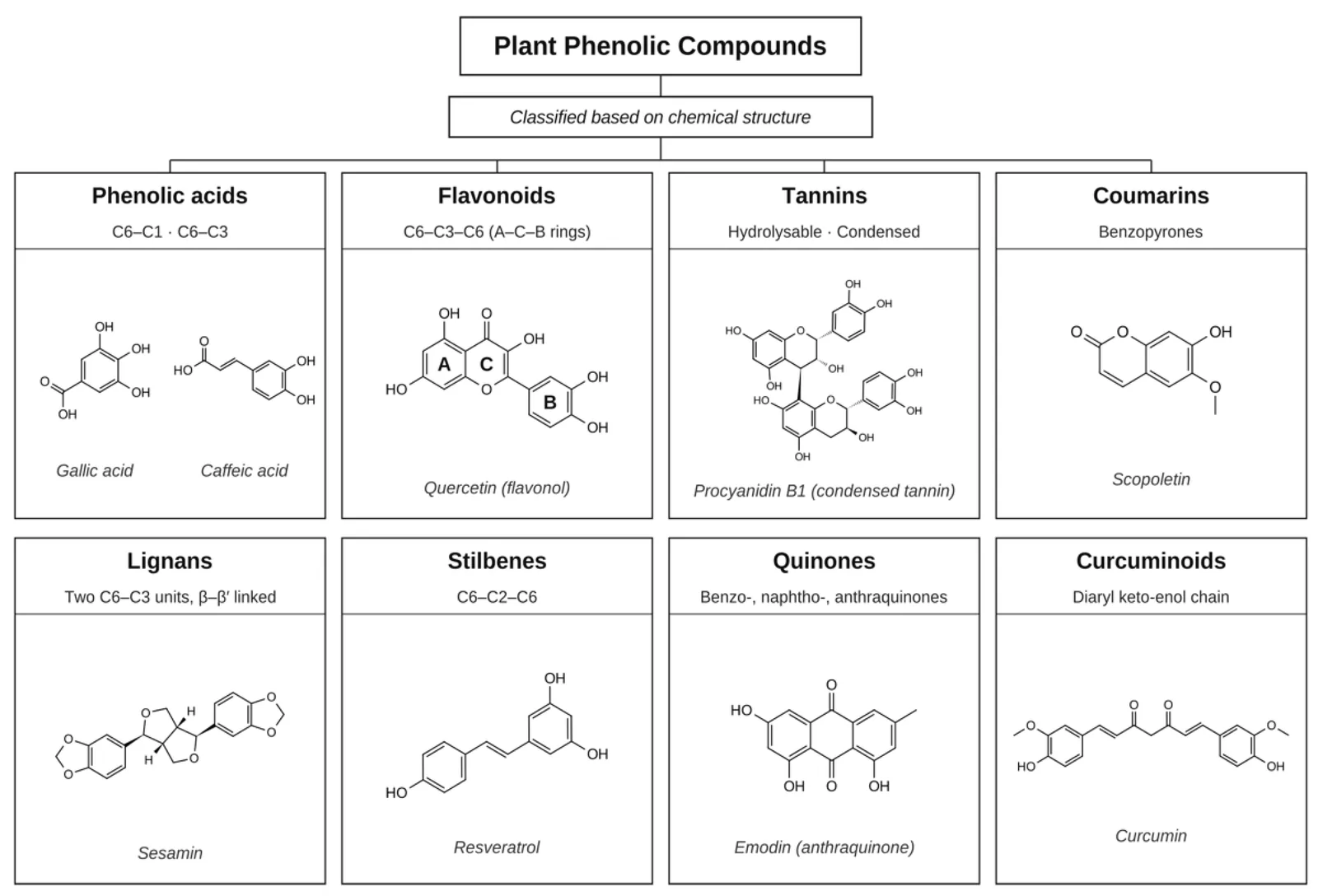
Classification of plant phenolic compounds based on chemical structure. The eight major classes are shown with their characteristic carbon skeleton or structural feature and a representative compound: phenolic acids (gallic acid, caffeic acid), flavonoids (quercentin), tannins (procyanidin B1), coumarins (scopoletin), lignans (sesamin), still bene (resveratrol), quinones (emodin) and curcuminoids (curcumin). In flavonoid structure, A, B and C denote the conventional ring designations of the C6-C3-C6 flavonoid skeleton: ring A is the benzene ring fused to heterocycle, ring C is the oxygen-containing heterocyclic (pyran) ring and ring B is the phenyl ring attached at C-2 ring C [18].

Plants can synthesize a wide variety of organic compounds that can be classified into two categories: primary and secondary metabolites. Primary metabolites are compounds involved in photosynthesis, respiration, growth, and development. Primary metabolites include phytosterols, acyl lipids, nucleotides, organic acids, and amino acids. The remaining phytochemicals, which accumulate in specific plant species, are called secondary metabolites [1]. Interest in understanding the significance of these secondary metabolites has grown, especially in their roles in protecting plants against different pests such as herbivores and microbial infections, attracting pollinators and seed-dispersing animals, acting as allelopathic agents, UV protectants, and signal molecules for nitrogen-fixing root nodules in legumes [2,3]. These secondary metabolites are typically derived from primary metabolite products; however, they have limited taxonomic distribution [4]. Plant phenolic compounds are classified based on the presence of one or more phenolic groups, which can vary in the number of hydroxyl groups and other substituents attached to them (Figure 1) [5]. They occur in plants in both free and conjugated forms. Phenolic compounds may include simple phenols and quinones, phenolic carboxylic acids and their derivatives, flavones, flavanols, catechins, and leukoanthocyans [5]. Phenolic compounds usually accumulate in the central vacuoles of guard cells, chloroplasts, and epidermal cells of leaves and shoots. Their accumulation levels in vacuoles increase upon exposure to light [6]. Some are linked covalently to the plant cell wall, while others occur in waxes or on the external surfaces of plant organs [7,8].

Plant phenolic compounds are generally grouped into several subclasses based on their chemical structure and biosynthetic origin [9]. The major groups include phenolic acids, flavonoids, tannins, lignins, stilbenes, and coumarins (Figure 1). Phenolic acids are further divided into hydroxybenzoic acids and hydroxycinnamic acids based on their carbon skeleton structure [10]. Flavonoids are considered one of the largest groups of phenolics and include flavones, flavonols, flavanones, anthocyanins, and isoflavones [11]. These compounds are widely distributed in fruits, vegetables, cereals and medicinal plants, and contribute to pigmentation, plant growth regulation, and provide protection against environmental stressors [12]. Tannins are high-molecular-weight phenolics that form complexes with proteins and play defensive roles against herbivores and microbial pathogens [13,14]. Lignins are important structural phenolics that strengthen the plant cell wall and contribute to water transport and mechanical support in plants [15]. The amount of a particular phenolic compound in plant tissue depends on the season and may also vary with growth and developmental stages. Internal and external factors such as trauma, wounding, abiotic stresses, and pathogen attack also affect their synthesis [16]. Photoinhibition, along with nutrient stresses such as deficiencies in nitrogen, phosphate, potassium, sulfur, magnesium, boron, and iron, can also trigger the synthesis of phenylpropanoid compounds in some plant species [6,17].

Phenolic compounds are mainly biosynthesized through the shikimate and phenylpropanoid pathways. The shikimate pathway is responsible for the production of aromatic amino acids, while the phenylpropanoid pathway converts phenylalanine into a variety of phenolic metabolites [19]. The key enzyme involved in this pathway is phenylalanine ammonia lyase (PAL), an important regulatory enzyme in phenolic biosynthesis [20]. Environmental stresses such as UV radiation, salinity, drought, pathogen infection, and heavy metal stress can influence PAL activity and increase the accumulation of phenolic compounds in plant tissues [21,22]. Increased phenolic synthesis under stress can help plants by modulating cellular redox homeostasis. Phenolics can limit excessive ROS accumulation and oxidative damage, while allowing ROS to function as important signaling molecules in stress and defense responses [23]. Phenolic compounds also play an important role in plant defense against biotic stresses. Plants are constantly exposed to different biological threats, including herbivorous insects, nematodes, and various plant pathogens, and therefore rely on several defense mechanisms to protect themselves. To resist such stressors, plants have developed specific physiological and metabolic pathways to synthesize a wide range of phenolic compounds [24,25]. These phenolic compounds act as protective agents, inhibitors, natural animal toxicants, and pesticides against invading organisms such as herbivores, nematodes, phytophagous insects, and fungal and bacterial pathogens [26,27]. Phenolic compounds such as phenolic acids, tannins, and resins can also deter birds by interfering with digestion through interactions with gut microflora [6,28]. In addition, phenolic compounds affect plant–plant interactions as allelochemicals present in all plant parts. For example, leaves contain *p*-hydroxybenzoic acid and *p*-coumaric acid, while bark and the rhizosphere feature quercetin, juglone, catechin, and sorgoleone. These compounds play a role in senescent leaves, in browning and pigmentation in flowers and fruits, and in pollination and seed dispersal. Some are linked to growth hormones like auxins (Indoleacetic acid, IAA). Monohydroxy B-ring flavonoids may act as cofactors for peroxidase, which destroys IAA, while dihydroxy B-ring forms inhibit IAA degradation [29,30].

Phenolic compounds are an important part of plant defense and contribute to resistance against a wide range of pathogens. Following pathogen infection, plants often increase the production and accumulation of phenolic compounds, which can restrict pathogen growth and strengthen plant tissues through cell wall reinforcement and lignification [8,26]. Beyond their antimicrobial properties, phenolics also participate in defense signaling pathways and activate localized and systemic resistance responses [26,31]. Variations in the composition and abundance of phenolic compounds among plant species and cultivars have been frequently associated with differences in disease susceptibility and resistance [8]. Therefore, understanding the diversity and functions of plant phenolic compounds is essential to elucidate plant–pathogen interactions and to develop sustainable disease management strategies. This review focuses on the major classes of plant phenolic compounds and their roles in plant defense against pathogenic microorganisms for major agricultural and horticultural crops.

## 2. Molecular Mechanisms of Phenolic-Mediated Plant Defense Responses

Phenolic compounds protect plants through multiple coordinated mechanisms, including direct antimicrobial activity, structural reinforcement of plant tissues, and indirect regulation of defense signaling pathways.

### 2.1. Direct Antimicrobial Activity

Many phenolic compounds act directly against fungal, bacterial, and nematode pathogens, functioning as antimicrobial compounds. There are preformed phenolic compounds, referred to as phytoanitcipins such as flavonols, dihydrochalcones, and phenolic acids. In contrast, phytoalexins are de novo products of responsive synthesis after pathogen attack in plants, such as flavonoids, glyceolin, medicarpin, coumestrol, and phaseolin. In rice, the flavonoid sakuranetin was the first identified phenolic phytoalexin, along with several phenylamide phytoalexins including *N-trans*-cinnamoyl tryptamine, *N-p*-coumaroyl serotonin, and *N-*cinnamoyl tyramine. These compounds directly inhibit bacterial and fungal pathogens of rice [32]. Root-secreted phenolic compounds also contribute to plant defense by suppressing harmful microorganisms while promoting beneficial microbial communities in the rhizosphere. Many phenolics, including flavonoids, glyceolin, medicarpin, coumestrol, and phaseolin, can disrupt microbial cell membranes and interfere with cellular metabolism, thereby limiting pathogen growth and colonization [33,34].

### 2.2. Structural Defense

Beyond the antimicrobial properties, phenolic compounds also contribute to plant structural defenses. A common response to pathogen invasion involves depositing phenolic compounds within the cell wall, strengthening the physical barrier against pathogen penetration [35]. Phenolic compounds also serve as precursors for lignin and suberin biosynthesis, which reinforce cell wall architecture and hence are sometimes associated with reduced fungal colonization and enhanced disease resistance. Lignification, a key plant defense response, involves complex three-dimensional deposition of lignin polymers within the cell walls. Resistant plants can initiate lignification more rapidly than susceptible ones. Lignification functions as a plant defense by creating a cross-linked barrier that strengthens the cell wall against pathogen invasion. Like salicylic acid (SA), lignin accumulation has also been associated with defense responses involving the hypersensitive response (HR). Lignin deposition can limit water and nutrient movement at infection sites and reduce cell wall permeability, thereby restricting pathogen growth and spread. For example, in *Colletotrichum orbiculare*—infected cucumber, lignin deposition at infection sites forms a physical barrier that restricts penetration by fungal appressoria. Similarly, in pepper plants exposed to *Phytophthora capsici*, rapid wall thickening is accompanied by the induction of acidic peroxidase isoenzymes and increased lignin polymerization. This localized lignification strengthens the cell wall and can form a physical barrier that restricts pathogen penetration and spread. Peroxidase-mediated oxidative reactions also contribute to the polymerization of phenolic precursors during lignin formation, further linking phenolic metabolism with cell wall-based defense. In *Asparagus officinalis*, roots inoculated with non-pathogenic *Fusarium oxysporum* induce systemic resistance; when later challenged with a highly pathogenic strain, these plants show faster, increased accumulation of lignin and defense enzymes, resulting in fewer necrotic lesions, a hallmark of nonhost resistance and systemic acquired resistance (SAR). Studies indicate that inhibiting lignin biosynthesis with chemical inhibitors makes resistant plants susceptible; for example, wheat plants resistant to *Puccinia graminis* lose their hypersensitive response when treated with lignification inhibitors. Similarly, inhibiting phenolic biosynthesis in barley increases susceptibility to powdery mildew [36,37,38,39,40]. Furthermore, the interaction between reactive oxygen species (ROS) and phenolic compounds is complex and extends beyond their roles as antioxidants and oxidants during plant defense responses. When a pathogen is recognized, controlled ROS production participates in defense signaling and may lead to oxidative coupling of phenolic compounds and to lignin formation in the cell wall. These reactions, which are in part carried out by peroxidases and laccases, help to strengthen the cell wall and thus can limit pathogen penetration and its spread. At the same time, phenolic compounds can affect the cellular redox balance, showing the dynamic relationship between ROS production, phenolic metabolism, and cell wall reinforcement during plant defense [41,42,43]. These studies suggest that phenolic compounds may indirectly contribute to disease resistance through lignin formation and cell wall reinforcement.

### 2.3. Regulatory and Signaling Roles

**Figure 2:**
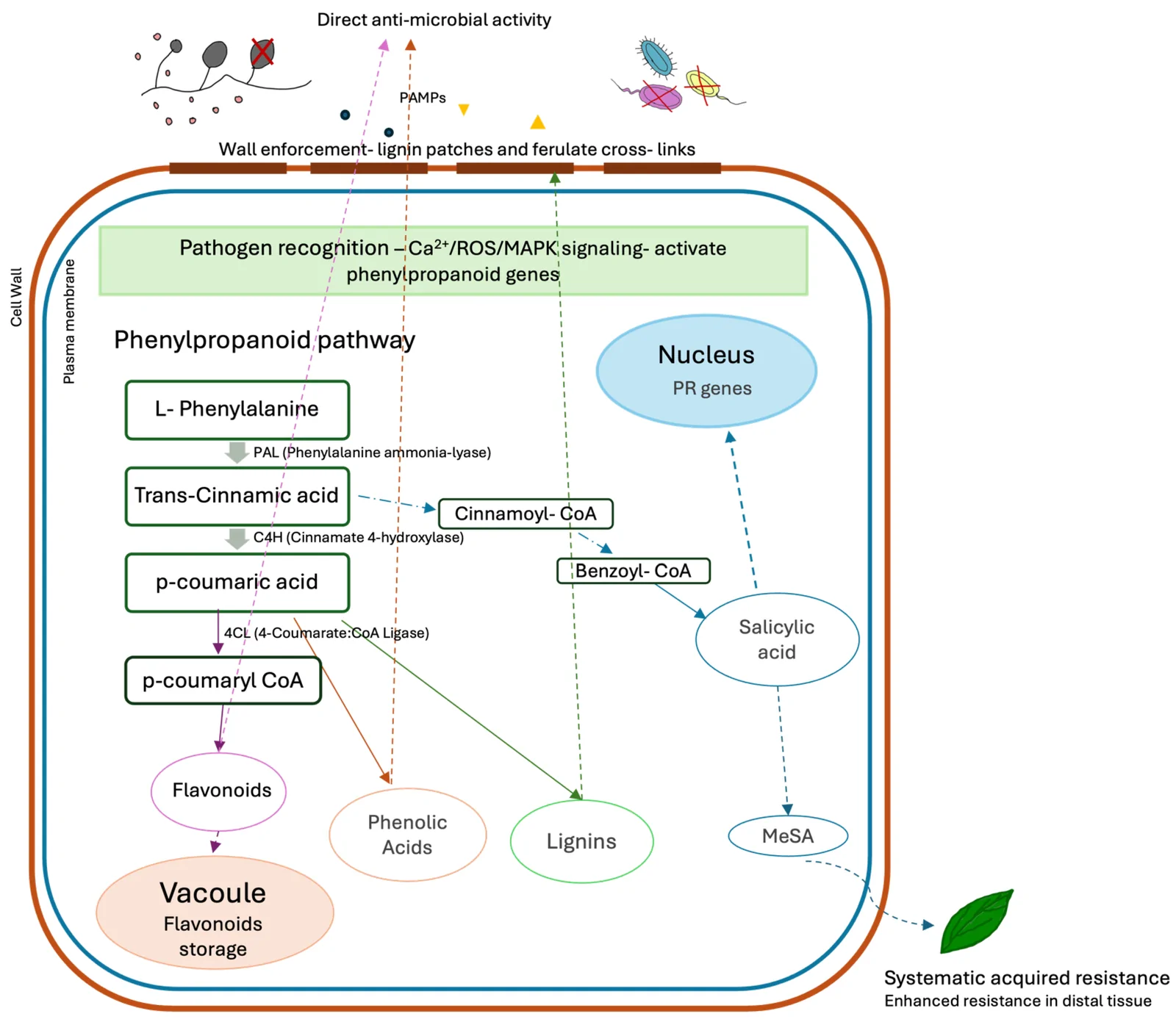
Phenylpropanoid pathway and phenolic defense outputs inside plant cells. Plant cells recognize pathogen-associated molecular patterns (PAMPs) from bacterial or fungal invasion, which initiates the signaling cascade through Ca^2+^ influx/reactive oxygen species (ROS) or mitogen-activated protein kinase (MAPK) pathways, leading to transcriptional activation in the nucleus, including the phenylpropanoid pathway. This pathway converts L-phenylalanine to p-coumarate using phenylalanine ammonia lyase (PAL) enzyme, leading to major phenolic outputs—flavonoids that act as direct antimicrobial compounds and are stored in vacuoles [47,48]; phenolic acids such as ferulic acid acting as antimicrobials and mycotoxin inhibitors [49]; lignins providing surface resistance by cross-linking cell wall; and a series of biochemical steps that convert trans-cinnamic acid into a major signaling compound. Salicylic acid [50] gets converted into methylated salicylic acid (MeSA) and contributes to Systemic Acquired Resistance (SAR) in distal leaf tissues. The solid arrow lines represent different types of Phenolics and dashed arrow lines represent mechanisms of resistant provided by those phenolic compounds.

Phenolic compounds also regulate plant immune responses, where basal resistance mechanisms such as cell wall reinforcement are activated during the early stages of plant defense, and phenylpropanoids and other phenolic compounds help strengthen structural barriers. Additionally, these compounds are also involved in induced resistance responses [44]. Plant immunity possesses multiple interconnected layers for defense, including pattern-triggered immunity (PTI) and effector-triggered immunity (ETI), which are initiated following recognition of pathogen-associated molecular patterns (PAMPs) and pathogen effector proteins, respectively, leading to the activation of defense responses such as ROS production, defense-related gene expression, and cell wall modifications [45,46] (Figure 2).

Although PTI and ETI are initiated through different recognition mechanisms, their downstream signaling networks can overlap and could mutually reinforce defense responses. Local immune activation can also trigger systemic signaling that contributes to establishing systemic-acquired resistance (SAR) in distal plant tissues. Pathogen-induced disruption of the plant cell wall activates phenylpropanoid metabolism, resulting in the accumulation of diverse defense-related metabolites. SA is a key signaling molecule and is closely integrated with PTI and ETI and helps regulate numerous defense responses. SA accumulation can promote the expression of defense-associated genes, including pathogenesis-related (PR) genes, through NPR1-dependent and NPR1-independent regulatory mechanisms [47,48,49,50,51,52] (Table 1).

SA biosynthesis begins in plastids from chorismate through two parallel pathways: isochorismate synthase (ICS) and PAL. PAL is a key enzyme in phenylpropanoid metabolism and can contribute to the accumulation of SA, phytoalexins, and other phenolic compounds associated with plant defense [51,52]. The relative contribution of these pathways varies among plant species. For example, both pathways contribute to SA production in soybean, whereas SA biosynthesis relies predominantly on the ICS pathway in *Arabidopsis* and the PAL-associated pathway in rice. *Arabidopsis* contains two ICS homologs, AtICS1 and AtICS2, with AtICS1 making the major contribution to SA production. Other enzymes and regulatory components, including AvrPphB SUSCEPTIBLE3 (PBS3), also influence SA biosynthesis [53,54,55]. Importantly, phenylpropanoid metabolism should not be considered solely as a pathway leading to the accumulation of antimicrobial phenolic compounds. Rather, it forms part of an interconnected defense network in which metabolic changes are linked with phytohormone signaling, production of defense-associated metabolites, cell-wall reinforcement, and regulation of oxidative responses. Phenylpropanoid biosynthesis begins with PAL and subsequently branches into pathways producing flavonoids, phenolamides, lignin precursors, and other defense-associated compounds [56,57] (Figure 2). Variation in these responses among plant genotypes is often associated with differences in disease resistance [56]. Through these interconnected processes, phenylpropanoid metabolism can participate in both biochemical and structural components of plant defense. Pathogens, in turn, can manipulate different components of this network, further illustrating the dynamic relationship between phenylpropanoid metabolism and plant immunity.

**Table 1:** Simplified functions of the genes associated with SA accumulation, signaling, and SAR.

Gene/Protein	Role in SA Signaling	Function	Reference
EDS1	Positive regulator of SA (SA accumulation)	Defense signaling related to PTI and ETI leads to SA accumulation	[58,59]
NDR1	Positive SA regulator	Required for PTI, ETI and SAR; function independent of EDS1	[60,61]
MOS, ALD1, EPS1	Positive regulator	Contributes to SA-mediated defense signaling	[62,63,64]
NPR1	Master regulator downstream of SA (activated by SA)	Controls PR gene expression and SAR	[65,66]
SSI1/SSI2/SSI4	NPR1-independent SA regulators (SA accumulation)	Constitutive SA accumulation and enhanced resistance	[67]

## 3. Counter-Defense of Pathogen

In response to phenolic-mediated plant defense, pathogens have evolved different strategies to overcome or tolerate these compounds, including their degradation, detoxification, or suppression of their production. Such counter-defense mechanisms can reduce the effectiveness of host defense and may contribute to the breakdown of resistance. The continuous interaction between plant defense and pathogen counter-defense therefore represents an important component of plant–pathogen coevolution [68]. Since phenolic-mediated defense responses are closely connected with other defense processes, pathogen counter-defense is also not limited to detoxification or direct degradation of phenolic compounds. Phenylpropanoid metabolism acts as an integrated network of SA signaling, ROS regulation, and direct antimicrobial metabolites. Pathogens can interfere with these interconnected defense processes at different levels, as discussed below:

(a) Suppression of phenylpropanoid transcription: Pathogens suppress gene transcription responsible for defense-related metabolites [69,70]. Highly aggressive pathogens like *Phytophthora infestans*, which causes Potato late blight (e.g., US-8 genotype), can significantly reduce the expression of key potato defense genes, such as *pal1* and *hmgr2*, leading to decreased phytoalexin levels [71,72]. Similarly, the fire blight pathogen *Erwinia amylovora* can block phenylpropanoid-related gene expression in susceptible apple genotypes, demonstrating that successful pathogens frequently target phenolic-mediated defense pathways to promote infection [73].
(b) Interference with hormone signaling: Some pathogens also interfere with key signaling pathways like SA, which is linked to phenylpropanoid metabolism. The phytotoxin coronatine (COR) produced by *Pseudomonas syringae* prevents SA accumulation by altering host gene transcription responsible for SA synthesis. COR can inhibit early physical defenses, such as callose deposition in roots [74,75].
(c) Detoxification of phytoalexins: Pathogens also detoxify and degrade existing phytoalexins, further weakening plant defenses. *P. sojae* and *P. infestans* secrete molecules like glucans, glucanase inhibitor proteins, and protease inhibitors (EPI1) to counteract plant defenses by suppressing protective phytoalexins [76,77,78]. The fungal pathogen *Mycosphaerella pinodes* produces specific defense suppressors during spore germination that inhibit phytoalexin synthesis in peas, and it has mechanisms to detoxify these suppressors.
(d) Oxidative-stress tolerance: Pathogens deploy stress-response regulators to tolerate host ROS, helping them overcome early defenses and suppress phenolic production. *Verticillium dahliae*, a soilborne pathogen causing wilt, can suppress SA-related defense genes (*Ha-PAL* and *Ha-NML1*) in sunflower, possibly using its necrosis- and ethylene-inducing protein to interfere with host responses [79]. Highly aggressive isolates express stress-response proteins such as SrrA, thioredoxin, and NADH-ubiquinone oxidoreductase to withstand host ROS toxicity [80,81].
(e) Effector-mediated manipulation: Many pathogens deploy effector proteins that suppress phenolic-associated defenses, including SA signaling and phytoalexin production. For example, *Pseudomonas syringae* suppresses phenylpropanoid pathway genes in *Arabidopsis thaliana* [82], while *Ustilago maydis* suppresses salicylic acid production in maize [83].

## 4. Interaction of Phenolic Compounds in Major Cropping Systems

Phenolic compounds receive significant attention in terms of their role in preventing diseases. Similar compounds can be found in cereal, fruit, and vegetable crops. In this section, we discuss the role of phenolic compounds in mediating plant–pathogen interactions in major cereals, fruits, vegetables, and legume crops.

### 4.1. Cereals

#### 4.1.1. Wheat (*Triticum aestivum* L.)

Phenolic compounds are important constituents of wheat metabolites and play a key role in wheat defense against pathogens. The enzyme PAL catalyzes the most important step of the phenylpropanoid pathway, linking primary metabolites into secondary metabolites with production of specialized metabolites. In wheat, PAL expression is rapidly induced following pathogen infection and contributes to the activation of phenylpropanoid-associated defense responses [84]. Important wheat pathogens that activate a coordinated phenylpropanoid response include biotrophic rusts such as *Puccinia striiformis* f. sp. *tritici*, and hemibiotrophic fungi such as *Zymoseptoria tritici* and *Bipolaris sorokiniana* [85,86]. In wheat, the phenolics could either be constitutive, providing a pre-existing chemical barrier against pathogen infection, or induced following pathogen infection. This constitutive accumulation occurs at higher rates in glumes, lemmas and palea of resistant plants than in susceptible ones. Induced defense-related metabolites include phenylamides such as cinnamoyl and coumaroyl conjugates of agmatine, putrescine, and tryptamine [87]. PAL is consistently induced during wheat host–pathogen interactions and is functionally required for resistance, as virus-induced silencing of *TaPAL* compromises wheat resistance to *Puccinia striiformis* f. sp. *tritici* (Pst) and reduces the accumulation of downstream lignin-related metabolites [87]. Among wheat diseases, researchers have evaluated the role of phenolic compounds most thoroughly in *Fusarium* head blight (FHB), caused primarily by *Fusarium graminearum* and *F. culmorum* [88]. For example, spikelet tissues of FHB-resistant cultivars (e.g., Frontana) show higher accumulation of free phenolics, particularly *p*-coumaric acids, after inoculation with *F. culmorum*, potentially contributing to pathogen resistance [89]. Moreover, in vitro as well as *in planta* studies showed that ferulic acid, the most abundant phenolic acid in wheat bran, efficiently inhibits type B trichothecene biosynthesis across multiple *Fusarium* species and chemotypes, which was observed to be associated with repressing the expression of *Tri* cluster genes [90]. Integrated metabolomic and proteomic profiling of near-isogenic lines carrying the major FHB-resistance QTL *Fhb1* further demonstrated that hydroxycinnamic acid amides, phenolic glucosides, and flavonoids of the phenylpropanoid pathway accumulated to higher levels in resistant wheat genotype lines after *F. graminearum* inoculation. These metabolites were associated with cell-wall thickening at the rachis node, which may contribute to restricting fungal spread [91]. In addition to FHB, phenylpropanoid-derived lignification also contributes to wheat defense against biotrophic rust fungi. Early inhibitor studies showed that treatment of resistant wheat with competitive inhibitors of PAL reduced lignification around necrotic host cells and was accompanied by greater spread of stem rust (*Puccinia graminis* f. sp. *tritici*) [39]. More thorough chemical analysis of the lignin actually deposited at sites of hypersensitive cell death in resistant wheat plants showed that it is enriched in syringyl units, suggesting compositional differences between defense-related lignin and constitutive cell-wall lignin [92]. For powdery mildew (*Blumeria graminis* f. sp. *tritici*), integrated transcriptome and metabolome profiling of resistant and susceptible wheat lines has shown that the flavone/flavonol biosynthesis branch is significantly induced upon infection, and that exogenous application of flavonoids to inoculated leaves reduces the size and number of mildew colonies, providing functional evidence that flavonoids contribute to wheat resistance against *B. graminis* [93].

Together, these studies establish that the constitutive and inducible phenolic metabolism in wheat, coordinated by the phenylpropanoid network, provides chemically diverse, multi-layered defense against a broad spectrum of pathogens, and that this metabolism is now a tractable target for both breeding and natural-product-based crop protection [49].

#### 4.1.2. Maize (*Zea mays* L.)

In maize, the seed pericarp plays a key role in resistance to ear-infecting pathogens, and the hydroxycinnamic-acid-rich primary cell wall provides the strongest constitutive phenolic defense [94]. Ferulic acid is the predominant phenolic compound in maize pericarp that is widely associated with maize genotypic resistance against pathogens [95]. A multi-genotype study found that dehydrodimers of ferulic acid (DFA) concentrations in the pericarp and aleurone tissues were negatively correlated with Gibberella ear rot caused by *Fusarium graminearum* disease severity, suggesting that higher DFA accumulation could potentially be associated with increased resistance [96]. Moreover, a 2-year field study on *F. verticillioides* causing ear and kernel rot of maize observed pericarp phenylpropanoids containing ferulic acid, p-coumaric acid, and diferulates with lower disease severity and reduced fumonisin (mycotoxin produced by the fungal pathogen) accumulation [97]. Similarly, another multi-genotype study confirmed the fumonisin-producing fungal species were significantly colonized at lower rates in genotypes with higher ferulic acid concentrations [97]. These secondary metabolites can also influence the biosynthesis of fumonisins [97]. For example, analysis of bioactive compounds at the milk dough stage has identified chlorogenic acid as the most abundant compound in the purified active fraction. This fraction showed up to 70% inhibition of fumonisin biosynthesis, suggesting that chlorogenic acid may contribute to the observed inhibitory activity [98]. The mechanistic fungicidal role of phenolics has also been established, suggesting that the antioxidant nature of many phenolic acids, including flavonoids and ferulic acid, can interfere with and modulate oxygenation in the process of the biosynthetic production of many mycotoxins, including fumonisins, by *Fusarium* species [49].

Along with *Fusarium* species, other important maize pathogens include the anthracnose pathogen *Colletotrichum graminicola* and the aflatoxin-producing fungus *Aspergillus flavus*. A high-throughput transcriptomic study showed that in *A. flavus*-infected resistant maize lines, flavonoid biosynthetic pathway genes were upregulated, and a significantly higher fold increase in the flavonoids naringenin and luteolin was found than in the susceptible line, suggesting an important contribution of flavonoids to plant defense mechanisms against aflatoxin contamination that might modulate the synthesis of toxins in maize kernels [99]. During maize-*C. graminicola* interactions, phenylpropanoid-related genes and *p*-hydroxycinnamic-acid-derived fluorescent material accumulate around infection sites. Thus, increased levels of phenolic compounds and lignin deposition following pathogen infection serve as one of the first barriers to infection by *C. graminicola.*

Taken together, studies in the past two decades have provided important evidence for the following conclusions: (1) cell wall associated ferulic acid and its derivated in the maize pericarp can be associated with plant resistance to ear-rot fungi and reduced mycotoxin accumulation, partly through their contribution to cell wall reinforcement; (2) soluble phenolic acids such as chlorogenic acid and other phenolic acids can suppress fumonisin biosynthesis by *F. verticillioides* by acting as antioxidants; (3) flavonoid and lignin biosynthesis contribute to maize defense against *A. flavus*, *C. graminicola*, and other fungal pathogens.

#### 4.1.3. Rice (*Oryza sativa* L.)

Rice deploys an extensive range of phenylpropanoid-derived phenolics such as phenolic acids, flavonoids, hydroxycinnamic acid amides, and lignin monomers as part of its chemical defense against fungal and bacterial pathogens. PAL, a key enzyme in the phenylpropanoid biosynthetic pathway, is encoded by a small gene family in rice. The role of the *OsPAL4* gene in disease resistance was demonstrated through genetic disruption of the gene, which substantially increased plant susceptibility to several bacterial and fungal pathogens, including *Magnaporthe oryzae*, *Xanthomonas oryzae* pv. *oryzae* (*Xoo*) and *Rhizoctonia solani*, providing direct genetic evidence that the *OsPAL4* gene contributes to broad-spectrum resistance [100]. In a separate study using a *T-DNA* knockout of *OsPAL06*, the mutant became more susceptible to *M. oryzae* invading from the roots and showed a near-complete loss of the flavonoid phytoalexins sakuranetin and naringenin, together with a roughly two-thirds reduction in root SA, providing strong evidence for phenolic compound-derived resistance against rice blast [101].

The flavanone sakuranetin is the most thoroughly characterized phytoalexin and a classic example of how phenolic chemistry contributes to fungal resistance among rice phenolic defensive metabolites [47]. Sakuranetin is biosynthesized from naringenin by the jasmonate-induced naringenin 7-*O*-methyltransferase [102]. For example, induced expression of the *OsNOMT* gene (naringenin 7-*O*-methyltransferase) increased sakuranetin accumulation 4-fold by the fourth day post-infection with the rice blast fungus *M. oryzae* [47]. This flavonoid was reported to interfere with clathrin-mediated endocytosis involved in the uptake of *M. oryzae* effectors, thereby limiting effector entry into host cells and contributing to rice blast resistance. This mechanism represents a distinct form of defense involving interference with pathogen effector uptake rather than the classical recognition and downstream immune signaling associated with many *R* genes [103]. Although sakuranetin was originally characterized for its resistance against blast, in vitro assays have shown that it also inhibits the growth of phylogenetically diverse rice pathogens, including *R. solani*, *Bipolaris oryzae*, and *Xoo*. In rice-*Xoo* interactions, overexpression of the jasmonate (JA)-responsive transcription factor *OsbHLH034* in infected plants up-regulated secretory peroxidase genes and significantly increased leaf lignin content, providing enhanced resistance to bacterial blight and demonstrating that JA-mediated reinforcement of the cell wall through lignification is a key plant defense mechanism [104]. Taken together, these studies indicate that phenolic metabolism contributes to multiple layers of rice defense. The PAL gene family supports phenylpropanoid metabolism, while phenolic phytoalexins (notably sakuranetin) and downstream lignin-biosynthetic enzymes contribute to multi-layered chemical and structural defense against rice’s principal pathogens.

#### 4.1.4. Other Cereal Crops

Beyond wheat, rice and maize, other three globally important cereal crops—barley (*Hordeum vulgare* L.), sorghum (*Sorghum bicolor* L. Moench.) and pearl millet (*Pennisetum glaucum* (L.))—all depend strongly on the phenylpropanoid pathway for resistance against their principal fungal and bacterial pathogens. For example, in barley, *Fusarium graminearum* is an important pathogen that causes FHB and, besides yield losses, produces several hazardous mycotoxins [105]. FHB-susceptible and resistant genotypes have been characterized for metabolic profiling, and multiple phenylpropanoid-related metabolites have been linked to provide resistance in barley, such as phenolic acids, flavonoid glycosides, and hydroxycinnamic acid amides (HCAAs) [106]. In sorghum, 3-deoxyanthocyanidins are important flavonoid phytoalexins associated with defense against the anthracnose pathogen *Colletotrichum graminicola*. Among these compounds, apigeninidin and luteolinidin are major 3-deoxyanthocyanidins that accumulate in response to pathogen infection and contribute to defense response [107]. In another sorghum-*Colletotrichum sublineolum* interaction study, resistant cultivars accumulated a diverse, complex mixture of phytoalexins including luteolinidin and 5-methoxyluteolinidin, which was absent in susceptible cultivars following fungal inoculation [108,109]. Compared with barley and sorghum, the role of phenolic compounds in pearl millet-pathogen interactions has been less extensively characterized. However, studies have shown that phenylpropanoid metabolism is associated with resistance to *Sclerospora graminicola*, the causal agent of downy mildew, an important disease of pearl millet. For instance, PAL activity was compared between downy mildew-resistant and susceptible pearl millet cultivars and was found to be higher in resistant cultivars. The highest PAL activity was detected in the actively growing region of young roots of resistant cultivars, supporting an association between increased phenylpropanoid pathway activity and downy mildew resistance [110]. Therefore, it has long been established that phenolic compounds play an essential role in cereals by providing defense against a diversity of fungal and bacterial pathogens. Further characterization of these compounds, the enzymes involved in their biosynthesis, and their underlying genes could provide valuable targets for breeding disease-resistant varieties and developing more sustainable approaches to disease management.

### 4.2. Legumes

Legumes (family *Fabaceae*) are the third-largest plant family, with more than 700 genera and 20,000 species. These, including major economically and ecologically significant crops such as soybeans, peas, and beans, provide the foundation for sustainable agriculture through their role as symbiotic nitrogen-fixing crops, thereby enhancing soil health. Although legumes are loaded with host-beneficial bacteria, they are also attacked by a myriad of microbial pathogens. To survive, legumes also regulate chemical defense responses, in addition to systemic or innate plant defenses. These specialized secondary metabolites fall into two categories: phytoanticipins, preformed antimicrobial compounds already present in plant tissue, and phytoalexins, synthesized in direct response to microbial invasion [111].

The Fabaceae family secretes secondary metabolites such as alkaloids, cyanogens, peptides, phenolics, polyketides, and terpenoids. In terms of phenolic diversity, legumes produce simple phenols (vanillin, syringic acid, ferulic acid, gentisic acid, gallic acid, p-hydroxybenzaldehyde); flavonoids (quercetin, kaempferol, etc.); isoflavones (genistein, daidzein, formononetin); pterocarpans (maackiain, glycinol, acanthocarpan, cristacarpin, glyceollin, medicarpin, phaseollin, pisatin, variabilin); catechins (catechin, epicatechin, catechin gallate, epigallocatechin gallate (EGCG)); tannins; and coumarins and furanocoumarins (umbelliferone, scopolin, psoralen, bergapten, xanthotoxin), all with antimicrobial or antifungal properties [3].

These metabolites have shown tissue-specific accumulation. Legumes largely secrete flavonoids, accumulating differently in the flowers and leaves, while some accumulate at higher levels in the seeds and roots. Soybeans produce both flavonols and isoflavones, with isoflavones accounting for 73–86% of total isoflavonoid production. Similarly, roots and seeds accumulate phenolic acids, flavonoids, betaines, and aldonic acids to facilitate selection of the Rhizobium community in the soil for the induction of nodulation (*nod*) genes [33].

In addition to their beneficial effects, isoflavones have been found to act as antimicrobials, enhancing resistance to various diseases. Soybean produces glyceollins and pterocarpans (medicarpin); pea produces pisatin; clover produces coumestrol; and bean produces phaseollins and phaseollinisoflavan [3]. Studies have found that within 24 h of Fusarium wilt exposure, isoflavonoids accumulated in legumes at high levels [112].

Phenolic biosynthesis in legumes follows the phenylpropanoid–flavonoid pathway. Early enzymes such as phenylalanine ammonia lyase (PAL), Cinnamate 4-hydroxylase (C4H), and 4-coumaroyl-CoA ligase (4CL) produce 4-coumaroyl-CoA. Chalcone synthase (CHS), along with Chalcone reductase (CHR) in legumes, forms chalcones. The legume-specific CHR converts naringenin chalcone to isoliquiritigenin, which feeds the isoflavonoid branch. Isoflavone synthase (IFS) carries out the key step, converting flavanones such as naringenin and liquiritigenin into isoflavones such as genistein and daidzein. Downstream, enzymes like isoflavone reductase (IFR), vestitone reductase (VR), and pterocarpan synthase convert isoflavones into pterocarpan phytoalexins, such as medicarpin. Genes for PAL, CHS, CHI, IFS, and IFR often appear in multigene families and are strongly activated by pathogens. For example, in Common Bacterial Blight-resistant beans, transcripts for PAL, C4H, CHS, and IFS increase within hours of *Xanthomonas axonopodis* infection, leading to the buildup of daidzein, genistein, and other phytoalexins [113,114]. Signaling molecules can help activate this process. For example, ethylene signaling is necessary for *Medicago truncatula* to synthesize isoflavonoids and resist *Rhizoctonia*. Similarly, salicylic acid treatments can induce the accumulation of daidzein, genistein, coumestrol, and phaseolin isoflavan in the Colombian *P. vulgaris* cultivar as a pathogen defense response [115]. In soybean, transcription factor GmMYB29A2 is essential for glyceollin I production and oomycete resistance. Silencing GmMYB29A2 abolished glyceollin I accumulation and rendered resistant soybean susceptible to *P. sojae*; conversely, overexpressing it in a susceptible cultivar boosted glyceollin I levels and restored resistance [116]. The resistant bean variety produced far higher levels of isoflavonoid phytoalexins (phaseollin, phaseollidin, and related compounds) in cotyledons and roots. Table 2 highlights the phenolic defense compounds identified in legumes following pathogen infection, along with their reported antimicrobial activities.

### 4.3. Forage Crops

Forage and fodder crops, including key legumes such as alfalfa (*Medicago sativa*) and clovers (*Trifolium* spp.), along with various forage grasses, are essential to global agriculture, providing the primary nutritional source for livestock. These crops support sustainable land use by improving soil structure and, in the case of forage legumes, fixing atmospheric nitrogen. Typically, alfalfa fodder contains three times more bound phenolic compounds than soluble ones, with the soluble extract mainly comprising catechin. At the same time, ferulic acid and *p*-coumaric acid predominantly make up the bound phenolics in leaves. In a study of alfalfa with varying susceptibility to *F. oxysporum*, susceptible seedlings had higher levels of free and cell-wall-bound phenolic acids, whereas resistant seedlings showed increases across all phenolic fractions (free, ester-, glycoside-, and cell-wall-bound) that contributed to higher total phenolic content. Cinnamic acid derivatives increased earlier and more markedly in resistant seedlings [117]. When infected by rust, alfalfa produces isoflavonoids, including coumestrol derivatives, that moderately inhibit fungi (approximately 40-60% growth inhibition at 50 µg/mL) and provide over 80% protection against wheat stripe rust at around 0.5 mg/mL [118]. The defensive role of fodder crop phenolics is well demonstrated by medicarpin’s function in alfalfa (*Medicago sativa*) when attacked by pathogenic fungi like *Rhizoctonia solani* and *Phoma medicaginis*. When these pathogens are detected, alfalfa quickly produces and releases medicarpin, which directly inhibits fungal germ tube growth and causes hyphal damage, confining the pathogen to the outer cortical cells and preventing vascular failure [119,120]. Likewise, in red clover (*Trifolium pratense*), the isoflavone formononetin plays a vital role in defending against *Sclerotinia trifoliorum*, a fungus responsible for crown and stem rot. Typically, formononetin is stored and secreted mainly as an inactive glycoside. When pathogen invasion damages tissues, host glycosidases quickly remove the sugar, releasing the potent aglycone form, which produces a localized, lethal antimicrobial burst at the point of fungal entry [111].

**Table 2:** Examples of phenolic defense compounds produced by plants in response to pathogen infection and their antimicrobial activities in legumes.

Crop	Organism	Disease	Molecule Compound	Effect	Reference
Soybean (*Glycine max*)	*Phytophthora sojae*	Phytophthora root and stem rot	Glyceollin	Inhibits mycelial growth and zoospore germination	[121]
Pea (*Pisum sativum*)	*Fusarium solani* f. sp. *pisi*	Root rot	Pisatin	Fungitoxic; limits pathogen spread	[122,123]
Alfalfa (*Medicago sativa*)	*Verticillium albo-atrum*/*Phoma medicaginis*	Verticillium wilt/Spring black stem	Medicarpin	Suppresses fungal colonization and growth	[124]
Chickpea (*Cicer arietinum*)	*Fusarium oxysporum* f. sp. *ciceris*	Fusarium wilt	Maackiain and Medicarpin	Inhibits spore germination and fungal development	[125]
Common bean (*Phaseolus vulgaris*)	*Colletotrichum lindemuthianum*	Anthracnose	Phaseollin	Strongly fungitoxic to mycelia and spores	[126]
Peanut (*Arachis hypogaea*)	*Aspergillus flavus*	Seed rot/Aflatoxin contamination	Resveratrol (Stilbenoid phenolic)	Antifungal activity; limits pathogen colonization	[127]
Red Clover (*Trifolium pratense*)	*Sclerotinia trifoliorum*	Sclerotinia crown and stem rot	Isoflavone formononetin	Antimicrobial activity against fungal hyphae	[128]
Soybean (*Glycine max*)	*Diaporthe phaseolorum* f. sp. *meridionalis*	Stem canker	Glyceollin	Accumulates as a primary defense mechanism against fungal attack, restricting pathogen growth	[129]
Pea (*Pisum sativum*)	*Nectria haematococca*	Root and stem rot	Pisatin	Functions as a toxic phytoalexin; successful pathogens must utilize specialized enzymes to detoxify it	[130]
Pigeonpea (*Cajanus cajan*)	*Fusarium udum*	Fusarium wilt	Cajanol	Strongly inhibits conidial germination and halts subsequent fungal growth at the site of infection	[131]

### 4.4. Vegetable Crops

Phenolic compounds are widespread throughout the plant kingdom and play diverse ecological roles, including important functions in vegetable–pathogen interactions.

#### 4.4.1. Tomato (*Solanum lycopersicum* L.)

Tomato is the second most widely cultivated vegetable crop globally and is grown under a range of climatic conditions. However, its adaptability also makes it vulnerable to various environmental stresses that favor infections caused by fungi, bacteria, viruses, phytoplasmas, and viroids. Among these, soil-borne diseases such as *Fusarium* wilt are particularly devastating and remain a major constraint to tomato production worldwide. Phenolic compounds have been shown to be influenced by biocontrol agents; for example, the biocontrol fungus *F. oxysporum* strain CS-20 enhances host-mediated resistance against vascular wilt caused by *F. oxysporum* f. sp. *lycopersici* by stimulating phenolic production [132]. Similarly, *Trichoderma harzianum* has been shown to increase overall phenolic content and activate defense-related genes in tomato plants when challenged with *Alternaria cerealis* MT808477 [133]. In contrast, some studies demonstrate that phenolic compounds can also act directly against pathogens. Exogenous application of phenolic-rich extracts, including gallic acid, chlorogenic acid, and rutin, has been reported to exhibit strong antifungal activity, with combined extracts showing synergistic effects [134,135]. These findings highlight the dual role of phenolics as both indirect defense regulators and direct antimicrobial agents.

Beyond these roles, phenolic compounds, particularly flavonoids, are involved in complex signaling networks during plant–pathogen interactions. For example, infection by *Pseudomonas syringae* Pst DC3000 induces the accumulation of phenylamides and salicylic acid through the SIMYB78 regulatory pathway, suggesting phenylpropanoid metabolism might be involved in bacterial defense responses [136]. Pathogens can also manipulate host metabolism to their advantage. Infection by tobacco mosaic virus alters the phenolic profile of tomato plants, increasing the accumulation of compounds such as rutin, ferulic acid, pyrogallol, and naringenin, while reducing levels of salicylic acid and chlorogenic acid, potentially facilitating viral replication [137]. A similar trend is observed during *Pythium aphanidermatum* infection, where reduced levels of phenolic acids like chlorogenic and ellagic acid correlate with disease progression [138].

Furthermore, phenolic compounds also contribute to resistance against postharvest diseases. In tomato fruit, volatile compounds released by beneficial bacteria can enhance resistance to gray mold caused by *Botrytis cinerea*. These volatiles trigger defense responses by modulating SA and JA signaling pathways [139]. Overall, accumulating evidence suggests that profiling phenolic compounds in tomato plants can provide valuable insights into disease resistance mechanisms. For example, resistant tomato cultivars exhibit higher levels of total phenolics and antioxidant compounds during early blight infection, supporting their potential use as markers in breeding programs [140].

#### 4.4.2. Potato (*Solanum tuberosum*)

Potato is the fourth most important food crop worldwide and represents a rich source of phenolic compounds, including phenolic acids and flavonoids such as flavanols and anthocyanins [141]. These compounds play a significant role in plant defense against a wide range of plant pathogens. Among these metabolites, chlorogenic acid is one of the most prominent phenolic compounds in potato and has been widely associated with antimicrobial activity and host defense responses. The accumulation of phenolic compounds can vary depending on the cultivar, as resistant potato varieties, particularly those showing resistance to soft rot caused by *Erwinia carotovora* subsp. *carotovora*, often exhibit higher levels of phenolics along with enhanced antioxidant activity. Furthermore, applying defense-related signaling molecules such as salicylic acid and methyl jasmonate has been shown to stimulate phenolic production, further highlighting their critical role in plant immune responses [142]. Multiple factors also influence the effectiveness of phenolic-mediated defense in potato, including pathogen type, leaf age, and cultivar-specific interactions. For instance, during infection by *E. carotovora*, basal (older) leaves tend to show higher enzyme activity but lower levels of phenolics and flavonoids, whereas younger leaves accumulate higher concentrations of these compounds, suggesting a dynamic and tissue-specific defense response [143]. Phenolic compounds also contribute to resistance against viral pathogens. For example, treatment with *Bacillus licheniformis* strain POT1 has been shown to induce the accumulation of multiple polyphenolic compounds and enhance the expression of flavonoid biosynthesis genes in response to Alfalfa mosaic virus infection [144]. Overall, phenolic compounds such as chlorogenic acid, flavonoids, and suberin-associated phenolics contribute to potato defense against diverse pathogens.

#### 4.4.3. Cucurbits

The Cucurbitaceae family is a large and diverse group comprising over 800 plant species distributed worldwide [145]. Similar to tomato and potato, cucurbits such as cucumber, squash, melon, and pumpkin produce a wide range of phenolic compounds, including flavonoids and phenolic acids, in response to phytopathogen attack. Phenolic compounds contribute to plant defense through both direct and indirect mechanisms. Some exhibit direct antimicrobial activity, whereas others participate in defense signaling, redox regulation, and structural defense. For example, during infection by *F. oxysporum*, the causal agent of Fusarium wilt in cucumber, the resistant lines showed greater accumulation of the flavonoid gallocatechin 3-O-gallate, suggesting an association between its accumulation and disease resistance [146]. In pumpkin, resistance to powdery mildew has been associated with increased phenolic content, lignin accumulation, and activation of phenylpropanoid pathway enzymes, all of which contribute to strengthening the cell walls [147]. Phenolic compounds also hold promise as environmentally friendly alternatives to synthetic pesticides. Certain phenolics, such as amentoflavone and quercetin, have demonstrated antiviral activity against Cucumber Mosaic Virus (CMV), suggesting their potential use in developing safer plant protection strategies [148]. However, plant–pathogen interactions involving phenolics are not always straightforward. Some pathogens can exploit these compounds as part of their counter-defense strategies. For instance, CMV infection in squash initially induces phenylpropanoid pathway gene expression but subsequently depletes phenolic compounds such as chlorogenic acid to facilitate systemic viral spread [149]. This dynamic interaction underscores the complexity of phenolic-mediated defense mechanisms and highlights their critical role in plant immunity. Beyond aboveground defense, phenolic compounds released by plant roots can also influence rhizosphere microbial communities. In cucumber monocropping systems, *p*-coumaric acid accumulation in soil or hydroponic environments has been shown to shift microbial communities toward phenol-degrading microorganisms. This shift can negatively affect plant growth, demonstrating that phenolics can also mediate antagonistic plant–microbe interactions under certain conditions [150].

#### 4.4.4. Other Root Vegetables

Phenolic compounds form an important component of the defense system in roots and storage organs of plants. Their dual role as antimicrobial agents and regulators of plant defense responses makes them particularly important in understanding belowground plant immunity. Carrot (*Daucus carota* L.), one of the most widely cultivated root vegetables globally, is rich in phenolic compounds, especially within the root periderm. These compounds occur primarily as hydroxycinnamic acids and their derivatives, with chlorogenic acid as the dominant phenolic metabolite. However, the concentration of these compounds can vary significantly by cultivar [151,152]. In addition to roots, carrot leaves are also a valuable source of antioxidant and antimicrobial compounds against bacterial pathogens such as *Pseudomonas aeruginosa*. These effects are largely attributed to phenolics, flavonoids, and glucosinolates, highlighting their potential as sustainable bioactive agents for disease management [153]. More examples of phenolic compounds with antimicrobial activity reported during plant–pathogen interactions in vegetable crops are summarized in Table 3. Beyond their direct antimicrobial functions, phenolic compounds can also serve as biomarkers for disease resistance. In crops such as sugar beet, resistant genotypes exhibit distinct metabolic profiles characterized by increased accumulation of specific phenolic derivatives and reduced levels of others. Comparable trends have also been observed in carrot and beetroot, suggesting that phenolic profiling could be a valuable tool for selecting disease-resistant varieties in breeding programs [154,155].

## 5. Challenges and Future Perspectives

Plant phenolic compounds are the most important classes of secondary metabolites involved in plant defense mechanisms against a wide range of pathogens, including bacteria, fungi, viruses, and oomycetes. Although considerable advances have improved our understanding of the biosynthesis, regulation, and antimicrobial properties of phenolic compounds, several challenges still limit their effective use for sustainable crop protection. One major challenge is the remarkable diversity and complexity of phenolic compounds. Plants produce thousands of structurally distinct phenolics, including phenolic acids, flavonoids, tannins, lignins, and stilbenes, each possessing unique biological activities and modes of action [21]. Genetic background, developmental stage, environmental conditions, and pathogen pressure influence the biosynthesis and accumulation of these compounds, resulting in substantial variation among plant species and even among cultivars of the same species [23,163]. Consequently, identifying specific phenolic compounds that consistently contribute to disease resistance across different pathosystems remains challenging.

An additional challenge is the lack of standardized methodologies for the extraction, isolation, identification, and quantification of phenolic compounds. Phenolic profiles reported in the literature can vary substantially depending on the solvent systems, extraction procedures, sample preparation methods, and analytical platforms employed [164,165,166]. This methodological variability complicates comparisons among studies and limits result reproducibility. Standardized protocols and analytical frameworks would improve data comparability and enable a more accurate assessment of the role of phenolics in plant defense.

The chemical complexity of phenolic profiles presents another significant limitation. Plant tissues contain intricate mixtures of phenolic compounds that interact with one another as well as with proteins, carbohydrates, and other metabolites, making it difficult to attribute specific biological activities to individual compounds [167]. Furthermore, although many phenolics exhibit direct antimicrobial activity, their role extends far beyond pathogen inhibition. Phenolic compounds contribute to cell wall reinforcement, modulation of reactive oxygen species (ROS), signaling pathways involving salicylic acid and jasmonic acid, and interactions with other defense-related metabolites and proteins [12,168]. Because these defense networks are highly interconnected, it is often difficult to determine the individual contribution of specific phenolics and their collective role in enhancing resistance. In addition, the molecular mechanisms underlying the biological activities of many phenolic compounds remain incompletely understood, limiting the ability to fully exploit their potential in plant protection strategies [12,169].

Translating laboratory findings into practical agricultural applications presents another important challenge. Most studies investigating phenolic-mediated defense responses are conducted under controlled environmental conditions, which may not accurately represent the complexity of agricultural production systems where plants are exposed to multiple abiotic and biotic stressors simultaneously [3,170]. Environmental factors such as temperature, water availability, soil characteristics, and microbial community composition can influence phenolic production, accumulation, and stability. Furthermore, increased allocation of resources to defense-related metabolites may impose metabolic costs that affect plant growth, reproduction, and yield, emphasizing the need to better understand the trade-offs between defense and productivity [171].

Despite these challenges, plant phenolic research continues to offer promising opportunities for improving sustainable disease management. Future studies should integrate advanced multi-omics approaches, including genomics, transcriptomics, proteomics, metabolomics, and phenomics, to provide a comprehensive understanding of phenolic-mediated defense mechanisms [172,173,174]. These technologies can help identify key regulatory genes, signaling networks, and metabolic pathways associated with enhanced resistance. In parallel, advances in metabolic engineering and genome-editing technologies such as CRISPR/Cas systems provide opportunities to manipulate phenolic biosynthetic pathways and improve disease resistance with greater precision than conventional breeding approaches [175]. However, because central phenylpropanoid genes are involved not only in defense but also in plant growth, development, structural integrity, and responses to environmental stress, manipulating them may cause pleiotropic effects and growth–defense trade-offs. Targeting key enzymes in the shikimate and phenylpropanoid pathways, including PAL, may selectively enhance the defense-related phenolic compounds in economically important crops.

Another promising area of future research involves understanding the interactions between phenolic compounds and plant-associated microbial communities. Growing evidence suggests that phenolics influence the composition and function of the rhizosphere microbiome and may contribute to microbiome-mediated disease suppression [176]. A better understanding of these interactions could lead to innovative strategies that combine host resistance mechanisms with beneficial microbial communities to improve crop health and resilience.

A broader principle emerging across crop-pathogen systems is that phenolic-mediated defense is highly context-dependent. The contribution of individual phenolics depends on their chemical properties, tissue and cellular localization, pathogen lifestyle, host genotype, developmental stage, and environmental conditions. Moreover, phenolic metabolism is closely linked to primary metabolism, growth, development, and other immune pathways, suggesting that enhancing one component of phenolic defense may involve physiological trade-offs. Thus, future research should prioritize approaches that combine targeted genetic and biochemical manipulation with spatially and temporally resolved measurements to distinguish causal defense mechanisms from responses that accompany infection.

## 6. Conclusions

As agriculture faces increasing pressures from climate change, evolving pathogen populations, and the need to reduce reliance on synthetic pesticides, plant phenolics represent an important component of plant defense with potential applications in sustainable crop protection. Across crop–pathogen systems, evidence supports several roles for phenolics in defense, including antimicrobial activity, cell wall reinforcement, modulation of reactive oxygen species and defense signaling, and regulation of both local and systemic immune responses. However, the strength of this evidence varies among phenolic compounds and defense mechanisms. Genetic, biochemical, and exogenous-application studies show that altering a specific phenolic compound or its biosynthetic or regulatory pathway can contribute to disease resistance. In contrast, the increased phenolic accumulation in resistant plants after pathogen infection remains largely correlative and does not, on its own, establish that a compound drives resistance. Harnessing these compounds in sustainable and integrated disease management programs may ultimately help reduce dependence on conventional agrochemicals while enhancing crop resilience. Continued interdisciplinary research across molecular biology, plant physiology, microbiology, ecology, biotechnology, and plant breeding will be essential to translate mechanistic knowledge into practical applications. Rather than treating phenolic accumulation itself as a universal marker of resistance, future disease-management strategies should focus on identifying and validating specific phenolic mechanisms that provide reproducible benefits across relevant crop-pathogen and environmental contexts. Such an evidence-based approach can support the development of resilient crop varieties and sustainable disease-management strategies while advancing long-term agricultural productivity and food security.

## Figures and Tables

**Table 3:** Examples of phenolic antimicrobial compounds produced during plant–pathogen interactions in vegetable crops.

Crop	Organism	Disease	Molecule Compound	Effect	Reference
Tomato (*Solanum lucopersicum*)	Not directly tested; Yeast and bacterial volatiles used as elicitors	Post-harvest diseases	Accumulation of phenylpropanoids, phenolic acids	Induced resistance against post-harvest diseases	[139,156]
Potato (*Solanum tuberosum*)	Not directly tested	DON (deoxynivalenol) used as an exogenous inducer	External treatment of DON leads to accumulation/changes in phenolic acids and flavonoids; activation of phenylpropanoid pathways	Changes in phenolic metabolism associated with defense response	[157]
Potato (*Solanum tuberosum*)	*Verticillium dahliae*	Verticillium wilt	Rutin (flavonoid)	Flavonoids involved in counter defense response	[147]
Pumpkin (*Cucurbita pepo*)	*Podosphaera xanthii*	Powdery mildew	Higher phenolics and activation of phenylpropanoid pathway	Higher phenolic accumulation was associated with greater resistance	[158]
Cucumber (*Cucumis sativus*)	*Botrytis cinerea*	Gray mold	Exogenous Nano-Se treatment induced changes in phenolic acid metabolism	Association with Induced resistance	[159]
Tomato (*Solanum lucopersicum*)	Tomato spotted wilt virus (TSWV)	Tomato spotted wilt	Quercetin, Kaempferol; Twi1-mediated glycosylation	Flavonoid glycosylation associated with defense against TSWV	[160]
Sugarbeet (*Beta vulgaris*)	*Cercospora beticola*	Cercospora leaf spot	Higher accumulation of Isovitexin derivatives in resistant germplasm	Genotypically resistant to plant	[161]
Carrot (*Daucus carota* subsp. *sativus*)	*Alternaria dauci*	Alternaria leaf blight	Higher levels of flavonoids -rutinosides in resistant accessions	Accumulation correlated with resistant genotypes	[162]

## Data Availability

No new data were created or analyzed in this study. Data sharing is not applicable to this article.
